# Transcriptome comparison revealed the difference in subcutaneous fat metabolism of Qinghai yak under different feeding conditions

**DOI:** 10.1371/journal.pone.0311224

**Published:** 2024-12-05

**Authors:** Weiqin Ding, Yonggang Sun, Yincang Han, Yaqian Liu, Shengwei Jin

**Affiliations:** 1 Academy of Animal Husbandry and Veterinary Sciences, Qinghai University, Xining, Qinghai, China; 2 Key Laboratory of Plateau Livestock Genetic Resources Protection and Innovative Utilization of Qinghai Provincial, Xining, Qinghai, China; University of Bologna, ITALY

## Abstract

In order to explore the differences in subcutaneous fat metabolism and pathway information in yaks under different feeding conditions, this experiment used Illumina high-throughput sequencing technology to sequence the transcriptome of subcutaneous fat tissues of yaks under different feeding conditions and analyzed them bioinformatically. 9 naturally grazed yaks at 18 months of age were randomly divided into 3 groups, one group (G18_SF) was slaughtered, one group (G24_SF) continued to graze until 24 months of age was slaughtered, and one group (F24_SF) was housed until 24 months of age was slaughtered, and subcutaneous fat tissue was collected from the back of the yaks. A total of 15,261 expressed genes were identified in the nine samples, with 13,959 coexpressed genes and 533 differential expressed genes (DEGs), G18_SF vs F24_SF 133 DEGs, G18_SF vs G24_SF 469 DEGs, F24_SF vs G24_SF 5 DEGs. GO functional annotation analysis found that DEGs were mainly annotated in BP and CC, which included biological regulation, metabolic processes and cellular processes. KEGG revealed that the DEGs are mainly enriched for PPAR signaling pathway, AMPK signaling pathway and other pathways related to lipid metabolism. This study provides a scientific basis for further research on the effects of mRNA on subcutaneous fat in yaks under different feeding conditions.

## Introduction

The yak of the Qinghai Plateau belongs to the order of even-toed ungulates, family Bovidae, and genus Bovine mammals, and is an indispensable dominant species on the Qinghai-Tibetan Plateau in the extremely neighboring areas, and is considered to be a source of livelihood and production for the local herdsmen. Yaks are considered to be an important part of livestock husbandry on the Tibetan Plateau, relying mainly on natural pasture grazing for their nutritional needs. Due to the unique geographic environment of the alpine pastoral area of the Tibetan Plateau, yak rearing is still primitive and natural grazing, and yak farming is greatly affected by natural conditions, especially the influence of the season [1]. In the period of dry grass, yaks have insufficient food intake, which results in energy that cannot satisfy their own needs, and fat metabolism is affected. Some studies have found that the growth of yaks can be improved after supplemental feeding during the dry grass period, so that they can pass through the dry grass period peacefully [2]. Therefore, house feeding of yaks during the dry grass period is favorable to the occurrence of their fat deposition.

Adipose tissue is an important energy metabolism organ of animal organism [3], which can be classified into subcutaneous fat (SF), intramuscular fat (IMF) and visceral fat (VF) according to its location, and the deposition of body fat is mainly concentrated in the subcutaneous fat tissue [4]. Fat deposition includes the processes of fat synthesis, transportation and catabolism. The main role of subcutaneous fat is to adiabatic and store energy, so the deposition of subcutaneous fat in yaks in winter plays an important role in their life. There are more studies on fat metabolism in chicken [5–8], duck [9–12], pig [13–15], etc., but there are fewer studies on fat deposition and metabolism in yak. In this study, we used transcriptome sequencing technology to screen and analyze the differentially expressed genes in subcutaneous fat on the back of yaks under different feeding conditions during the withering grass period, and analyzed the KEGG enriched pathway of the differential genes, aiming to excavate the genes related to fat deposition of yaks in Qinghai from molecular point of view, and to provide references for the study of fat metabolism of yaks in Qinghai.

## Materials and methods

### Experimental animal and ethics

Nine 18-month-old male yaks of natural grazing, similar weight and good physical condition were selected from the Meilongzhang Animal Husbandry Professional Cooperative in Qilian County, Haibei Tibetan Autonomous Prefecture, Qinghai Province, China. They were randomly divided into three groups. At the beginning of the experiment in October, three yaks were slaughtered (G18_SF) to serve as the control group (n = 3). The other two groups, one group grazing naturally for 6 months to 24 months old (G24_SF) on the same pasture. The other group (Gansu Minle, China) was fed fattening for 6 months to 24 months old (F24_SF) in the barn, and after the test was completed, the subcutaneous fat on the back of the test was collected, and the samples were transported back to the laboratory in a liquid nitrogen tank, and stored at -80°C until subsequent use.

The guillotine method renders the yaks rapidly unconscious, there were no chemicals to contaminate biological tissues. The whole process was as quick as possible to alleviate the animal’s suffering. All animal procedures for experiments were approved by the Committee of Experimental Animal Care, and Handling Techniques were approved by Qinghai University, China.

### Feeding management

Yaks in the natural grazing group had unrestricted access to graze in the same pasture from 07:00 to 18:00 daily, allowing them to freely eat and drink during this period. The grazing pasture primarily consisted of *Stipa capillata L*, *Poa annua*, and *Artemisia argyi*, with the nutrient levels of the forage detailed in Table 1. On the other hand, yaks in the whole-housed group were fed using the TMR (total mixed ration) mode. The composition and nutrient levels of the basal ration can be found in Table 2. These yaks were fed once at 07:00 and again at 17:00, with the concentrate provided in pellet form from a feed mill located in Minle County, Gansu Province, China.

**Table 1:** Forage nutrient levels (%).

Items	Moisture	Crude Protein	Ether extract	Coarse Ash	CF	ADF	NDF	Ca	P
Grass Period	26.01	9.96	2.55	15.79	29.75	34.81	46.35	2.82	0.14
Withered Period	11.16	7.06	1.33	21.77	38.25	53.31	66.47	1.10	0.04

**Table 2:** Basal diet composition and nutrient levels (dry matter basis).

Items	Proportion (%)	Nutrientl levels	Content
Corn	43.71	Combined net energy (Mj/kg)	4.28
Concentrate	10.93	Crude protein	16.63
Rapeseed meal	3.0	Ether extract	5.49
Soybean meal	3.0	Crude fiber	21.17
Oat hay	10.93	Acid detergent fiber	28.61
Silage	27.33	Nutral detergent fiber	46.09
Salt	0.55	Calcium	0.36
NaHCO3	0.55	Total phosphorus	0.16
Total	100		

### Total RNA extraction and cDNA synthesis

The total RNA from an appropriate amount of fat tissue was extracted using the total RNA extraction kit (Beijing Tiangen, China) according to the instructions. The concentration and purity of total RNA were determined using NanoDrop One. Total RNA was reverse transcribed into cDNA using reverse transcription kit (Tiangen, Beijing, China) and stored at -20°C.

### Sequencing data quality control and sequence comparison analysis

Raw datas from each sample were subjected to sequencing-related quality assessment using fastp. Seqprep (https://github.com/jstjohn/SeqPrep) was performed to obtain high-quality QC data (Clean reads) for subsequent analysis. The clean reads after quality control were compared with the reference genome (LU_Bosgru_v3.0) to obtain mapped data (reads) using Hisat2 (https://ccb.jhu.edu/software/Hisat2/index.shtml) for subsequent transcript assembly, expression calculation, etc. The reconstruction of transcripts was carried out using Stringtie software (http://ccb.jhu.edu/software/stringtie/).

### Screening, functional annotation and enrichment analysis of differentially expressed genes

Differently expressed products conditioned on Padjust < 0.05& |log2 Fold change | ≥ 1 were screened by DESeq2 [16] difference analysis software based on binomial distribution, Padujst adjustment method for Benjamini and Hochberg (BH), Subsequently, differentially expressed genes (DEGs) were analyzed by Gene Ontology (GO) and Kyoto Encyclopedia of Genens and Genomes (KEGG) functional enrichment analysis. The analysis of Padjust < 0.05 for this experiment was determined to be significantly enriched.

### Real-time fluorescence quantitative validation

Three genes were randomly selected from the differential genes, and the accuracy of transcriptome sequencing was verified by qRT-PCR. The 2^-ΔΔCt^ method was used for the calculation of the expression between samples, and the relative expression was statistically analyzed by t-test, and the results were expressed as “Mean ± Standard Deviation (Mean ± SD)”. Primer information is shown in Table 3.

**Table 3:** Primer information.

Name	Sequence (5,-3,)	Length
ROBO2	F:TGTATTGCGGAGAATCGGGTTG R:TCACTGTTCGACCTTGAGCAAC	130
ITSN1	F: TGGTGGAAGGGTGAAGTCCA R: TAAGTCTGAGCACCATTGCTGG	102
MMP19	F: GGAGGGCGCAAACTCTGAAG R: TTGCAACAAGTAATCCACAGC	143
GAPDH	F:AGTTCAACGGCACAGTCAAGG R:ACCACATACTCAGCACCAGCA	124

Note: *ROBO2*: roundabout guidance receptor 2, *ITSN1*: intersectin 1, *MMP19*: matrix metallopeptidase 19, *GAPDH*: glyceraldehyde-3-phosphate dehydrogenase.

## Result

### Quality analysis of RNA-Seq data

The quality of total RNA extracted from yak subcutaneous fat tissue was examined before sequencing, and the results showed that the concentration of RNA was greater than 30 ng/μL, and the A260/A280 ranged from 1.8 to 2.0, which indicated that the RNA samples of the present experiment were of high purity and good integrity, and they met the requirements of sequencing, and could be carried out for the subsequent experiments. In this experiment, I created a total of 9 libraries named G18_SF1, G18_SF2, G18_SF3, F24_SF1, F24_SF2, F24_SF3, G24_SF1, G24_SF2, G24_SF3. After sequencing on the Illumina sequencing platform, the original reads ranged from 112682658 to 134611426 (Table 4). After excluding low-quality sequences, there were more than 111,875,262 clean reads per sample. The error rate of the nine sequencing libraries was 0.025%. The Q20 and Q30 of the 9 sequencing libraries were 98.10% and 94.50% respectively. Overall, the quality of the sequencing data was good and met the requirements of our follow-up experiments.

**Table 4:** Statistics of mRNA sequencing data.

Sample	Raw Reads	Clean Reads	Error rate(%)	Q20(%)	Q30(%)	GC content (%)
G18_SF1	118092066	117132478	0.0239	98.41	95.29	48.26
G18_SF2	120252036	118675150	0.024	98.38	95.26	48.26
G18_SF3	117336804	116135638	0.0242	98.29	95.05	49.46
F24_SF1	121904678	121149048	0.0246	98.19	94.58	47.96
F24_SF2	126380884	125486926	0.0246	98.16	94.58	48.31
F24_SF3	117849858	116880732	0.0246	98.15	94.57	51.36
G24_SF1	134611426	133828336	0.0244	98.26	94.8	49.91
G24_SF2	126427892	125636990	0.0247	98.14	94.45	47.78
G24_SF3	112682658	111875262	0.0245	98.19	94.65	49.5

### Sequencing data comparison and analysis

Compare the clean reads with the Yak reference genome to obtain the map reads (Table 5) required for subsequent transcript assembly and expression calculations, and evaluate the quality of the comparative transcriptome sequencing results. The Total reads of all 9 samples are above 110,000,000, Total mapped are above 92%, Multiple mapped are between 10% and 16%, and Unique mapped are around 80%.

**Table 5:** mRNA sequence alignment results.

Sample	Total reads	Total mapped	Multiple mapped	Unique mapped
G18_SF1	117132478	109277393(93.29%)	12808172(10.93%)	96469221(82.36%)
G18_SF2	118675150	109718719(92.45%)	13380817(11.28%)	96337902(81.18%)
G18_SF3	116135638	108023657(93.02%)	14107563(12.15%)	93916094(80.87%)
F24_SF1	121149048	111828271(92.31%)	12865848(10.62%)	98962423(81.69%)
F24_SF2	125486926	116775300(93.06%)	13514201(10.77%)	103261099(82.29%)
F24_SF3	116880732	108065445(92.46%)	18392131(15.74%)	89673314(76.72%)
G24_SF1	133828336	123208623(92.06%)	16401001(12.26%)	106807622(79.81%)
G24_SF2	125636990	117409963(93.45%)	13096537(10.42%)	104313426(83.03%)
G24_SF3	111875262	104283247(93.21%)	14230090(12.72%)	90053157(80.49%)

### Gene expression analysis

**Fig 1:**
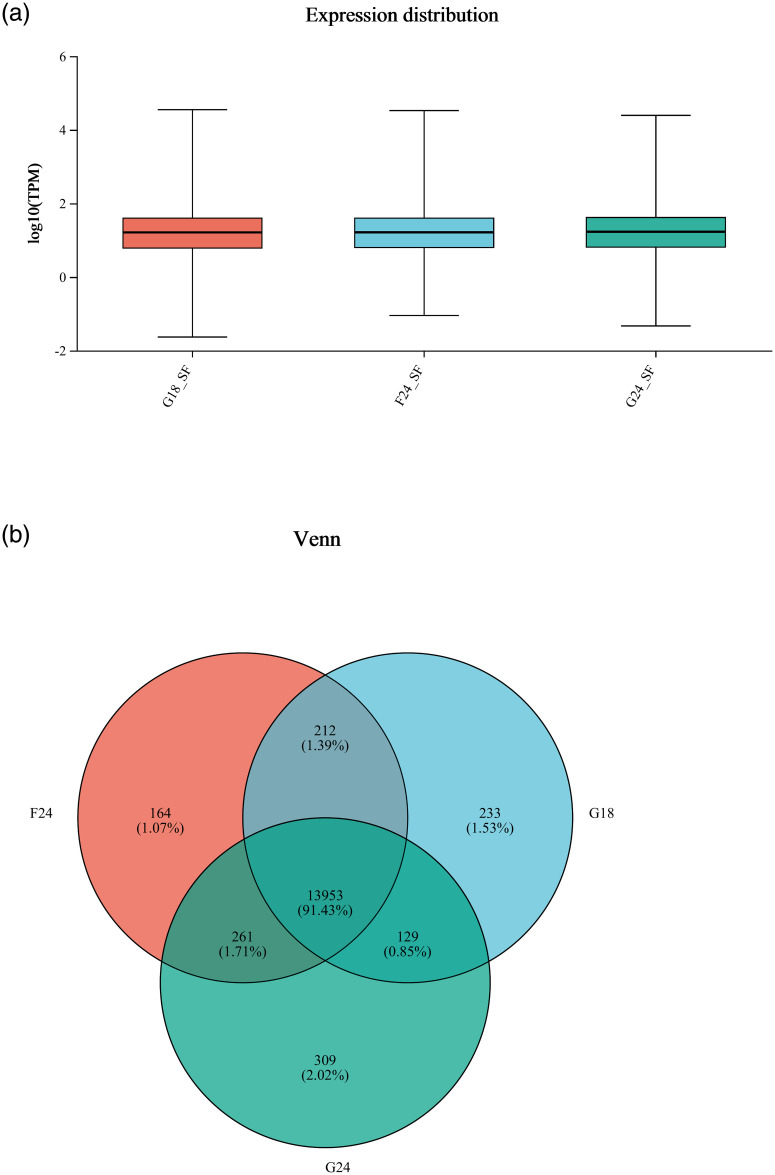
Box plots and Venn. a: Box plot of expression distribution, b: Inter-sample Venn diagram.

To better understand the differences between the groups, we performed an expression analysis of all (known and novel) genes in the three experimental groups. According to the box plot of TPM distribution, the gene expression distributions of the three experimental groups were similar (Fig 1a). A total of 15,261 expressed genes were identified in the three experimental groups, of which 13,959 were co-expressed, G18_SF had 233 unique genes, G24_SF had 309 unique genes, F24_SF had 164 unique genes. There are 129 genes common to G18_SF and G24_SF. There are 212 genes common to G18_SF and F24_SF. There are 261 genes common to F24_SF and G24_SF (Fig 1b).

**Fig 2:**
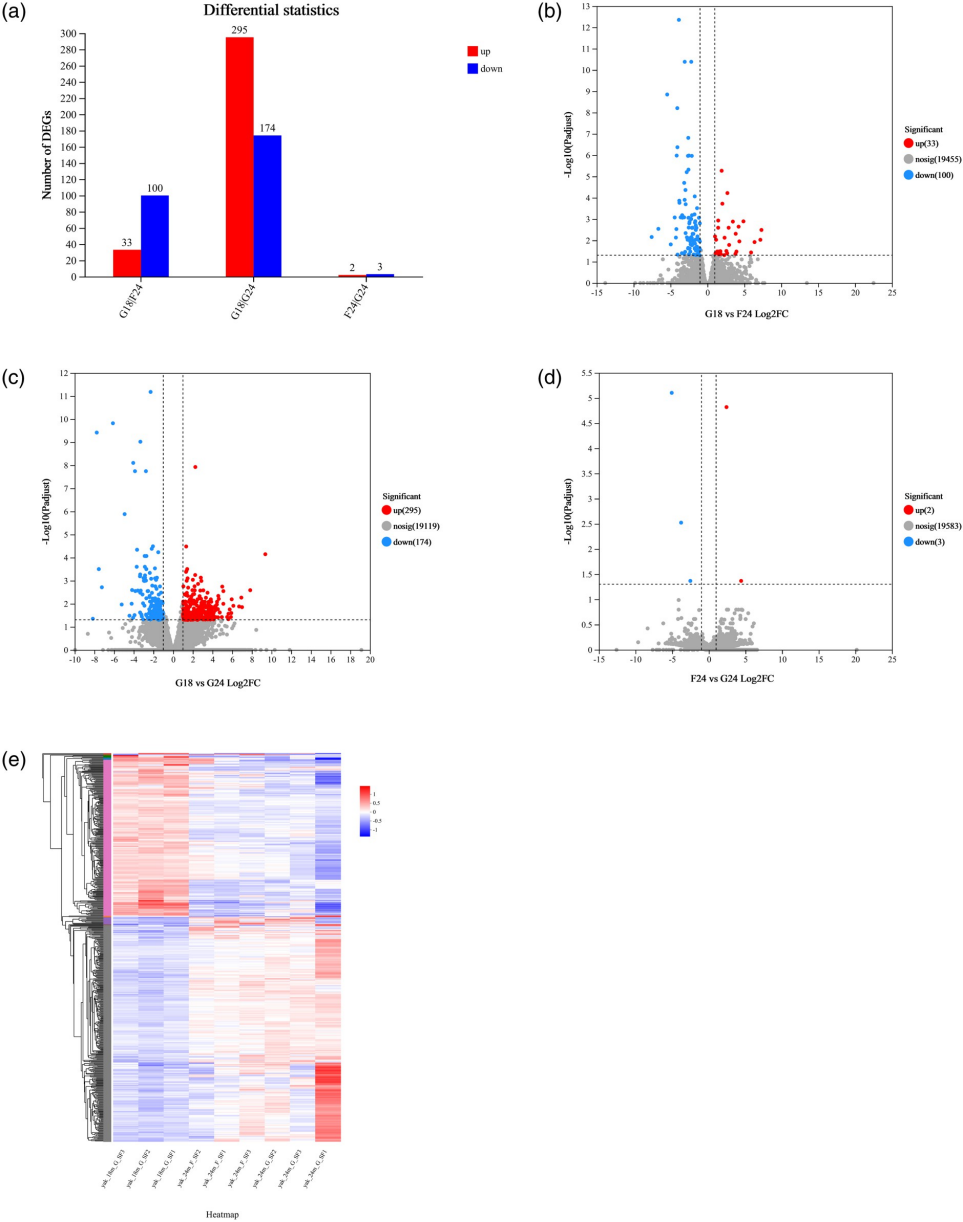
Differently expressed genes and clustering heat map. a: Histogram of differentially expressed genes, b, c, d: Volcano map of differentially expressed genes, e: Heatmap of clustering between samples of differentially expressed genes.

In this experiment, all DEGs of yak subcutaneous adipose tissue from different feeding methods were screened with Padjust <0.05& |log2FC| ≥1, and 533 differential expression genes (DEGs) were screened. A total of 133 DEGs were identified by comparison of G18_SF and F24_SF, of which 33 were up-regulated and 100 were down-regulated; a total of 469 DEGs were identified by comparison of G18_SF and G24_SF, of which 295 were up-regulated and 174 were down-regulated; and a total of 5 DEGs were identified by comparison of F24_SF and G24_SF, of which 2 were up-regulated and 3 were down-regulated (Fig 2a–2d). In addition, to further understand the differential gene expression patterns of the three groups, the clustered heatmap (Fig 2e) analysis visualized the changes in the differential gene expression of each experimental group versus the control group. The expression patterns of differential genes within each group of were similar.

### GO functional annotation analysis

**Fig 3:**
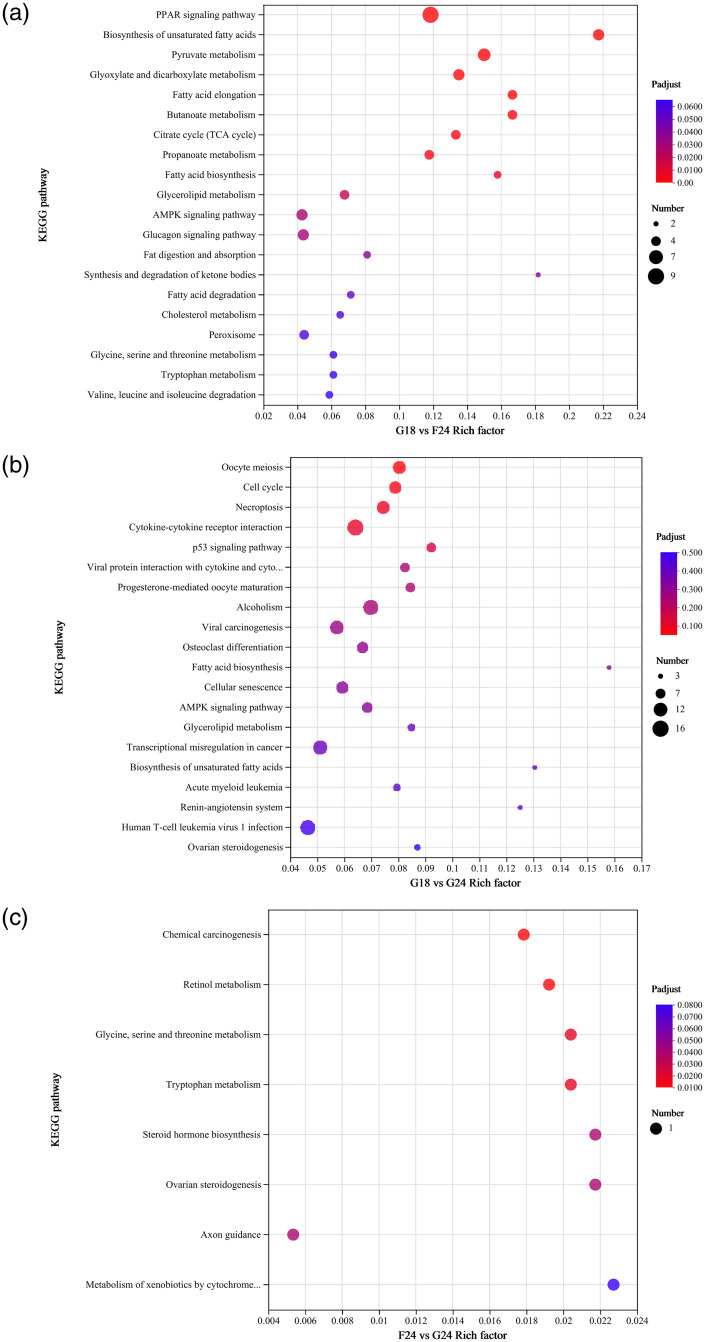
GO functional enrichment analysis of DEGs.

To further understand the function of DEGs in subcutaneous fat tissue, GO functional annotation analysis was performed on DEGs from three control groups, and the results are shown in Fig 3. Similar to existing studies, DEGs were enriched into 3 main categories, including biological_processes (BP), molecular_function (MF) and cellular_component (CC). Annotation results based on the GO database showed that 133 DEGs were significantly enriched into 18 BPs, 14 CCs, and 10 MFs in G18_SF vs F24_SF. GO entries function in processes such as bioregulation, metabolic processes, cellular processes, catalytic activity, molecular construction, and organ and cell parts. In addition, DEGs were also enriched for processes such as stimulus responses. 469 DEGs were significantly enriched in 21 BPs, 16 CCs, and 11 MFs in G18_SF vs G24_SF. GO entries function mainly in processes such as metabolic processes, bioregulation, cellular processes, catalytic activity, molecular construction, protein complex encapsulation, membranes, membrane fractions, organ fractions, and cellular fractions. There are also processes such as biological localization, developmental processes, cellular composition, tissue or biogenesis, and stimulus response. In F24_SF vs G24_SF, 5 DEGs were significantly enriched in 10 BPs, 7 CCs, and 2 MFs. Mainly annotated to cellular processes and molecular constructs sections. There were also membrane, cellular part, and membrane part. The GO functional annotation analysis revealed that DEGs were mainly annotated in BP and CC, which showed that BP and CC were the main biological functions exercised by DEGs from subcutaneous fat of yaks with different feeding methods during the withering period.

### KEGG functional enrichment analysis

**Fig 4:**
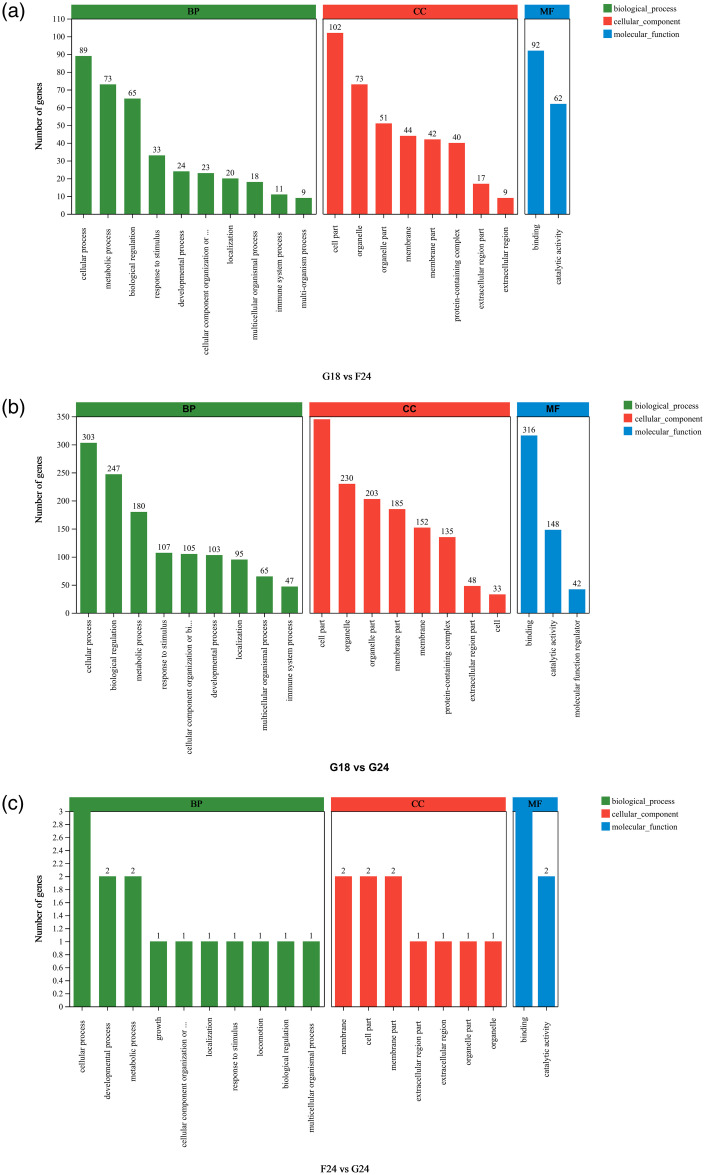
KEGG enrichment analysis.

To further analyze the pathways that may be enriched by DEGs screened in subcutaneous fat tissue, KEGG enrichment pathway analysis was used to further analyze DEGs.The results of the first 20 enriched pathways for each comparison group are shown in Fig 4. 101 out of 133 DEGs in G18_SF vs F24_SF were able to be enriched by KEGG, and DEGs were enriched to 209 pathways, of which 37 were significantly enriched (*P*<0.05). The KEGG pathway most significantly enriched by DEGs was the PPAR signaling pathway (*P*<0.01), and nine genes were enriched to this pathway, including *LPL* and *SCD*. 371 out of 469 DEGs in G18_SF vs G24_SF were able to be enriched by KEGG, and DEGs were enriched into 281 pathways, with 26 pathways significantly enriched (*P*<0.05). The KEGG pathway most significantly enriched by DEGs was Alcoholism (*P*<0.05). 8 genes such as *FASN*, *LEP*, and others were significantly enriched to the AMPK signaling pathway (*P*<0.05). *ACACA*, *ACSL1* and *FASN3* genes were significantly enriched for Fatty acid biosynthesis (*P*<0.05). Three of the five DEGs in F24_SF vs G24_SF were KEGG-enriched, and DEGs were enriched for eight pathways, with the most significant KEGG pathways being Metabolism of xenobiotics by cytochrome P450, Steroid hormone biosynthesis, and Steroid hormone biosynthesis. Hormone biosynthesis, and Ovarian steroidogenesis (*P*<0.01), and one study found that Ovarian steroidogenesis is associated with lipid metabolism. Five significant pathways related to adiposity were randomly selected in each control group, and genes related to lipid metabolism and muscle development, such as *FASN*, *SCD*, *ACSL1*, and *CYP1A1*, were screened according to the significant pathways (Table 6).

**Table 6:** Some KEGG pathways related to lipid metabolism are present.

group	Pathway id	Description	pvalue	gene	regulate
G18_SFvsF24_SF	map03320	PPAR signaling pathway	3.45E-09	*ACSL1、ACOX2、EHHADH、SCD、LPL*	down
				*ANGPTL4、APOA1*	up
	map01040	Biosynthesis of unsaturated fatty acids	5.31E-07	*TECR、SCD、ELOVL5、ELOVL6、HSD17B12*	down
	map00062	Fatty acid elongation	2.40457E-05	*TECR、ELOVL5、ELOVL6、HSD17B12*	down
	map00071	Fatty acid degradation	0.003389096	*EHHADH、ACSL1*	down
	map04152	AMPK signaling pathway	0.001549517	*PFKFB1、SCD、ACACA、FASN*	down
				*CREB3L3*	up
group	pathway id	description	pvalue	gene	regulate
G18_SFvsG24_SF	map04152	AMPK signaling pathway	0.012426127	*FOXO1、LEP、FASN、ACACA、SCD*	down
				*PPP2R1B、CCNA2、CREB3L3*	up
	map00061	Fatty acid biosynthesis	0.012968177	*ACACA、ACSL1、FASN*	down
	map04060	Cytokine-cytokine receptor interaction	0.001023328	*LIFR、TNFRSF19、LEP*	down
				*IFNGR2、IL7R、TNFSF13、EPOR、CCR2、IL17RB、CCR5、TGFB1*	up
	map01040	Biosynthesis of unsaturated fatty acids	0.021931698	*SCD、HSD17B12、ELOVL6*	down
	map04913	Ovarian steroidogenesis	0.032812823	*CYP1A1、ADCY5*	down
				*ALOX5*	up
group	pathway id	description	pvalue	gene	regulate
F24_SFvsG24_SF	map04913	Ovarian steroidogenesis	0.009802459	*CYP1A1*	down
	map05204	Chemical carcinogenesis	0.011924916	*CYP1A1*	down
	map00140	Steroid hormone biosynthesis	0.009802459	*CYP1A1*	down
	map00260	Glycine, serine and threonine metabolism	0.010439515	*PSPH*	up
	map04360	Axon guidance	0.039449611	*SLIT1*	down
group	pathway id	description	pvalue	gene	regulate
F24_SFvsG24_SF	map04913	Ovarian steroidogenesis	0.009802459	*CYP1A1*	down
	map05204	Chemical carcinogenesis	0.011924916	*CYP1A1*	down
	map00140	Steroid hormone biosynthesis	0.009802459	*CYP1A1*	down
	map00260	Glycine, serine and threonine metabolism	0.010439515	*PSPH*	up
	map04360	Axon guidance	0.039449611	*SLIT1*	down

Note: *ACSL1*: acyl-CoA synthetase long chain family member 1, *ACOX2*: acyl-CoA oxidase 2, *EHHADH*: enoyl-CoA hydratase and 3-hydroxyacyl CoA dehydrogenase, *SCD*: stearoyl-CoA desaturase, *LPL*: lipoprotein lipase, *ANGPTL4*: angiopoietin like 4, *APOA1*: apolipoprotein A1, *TECR*: trans-2,3-enoyl-CoA reductase, *ELOVL5*: ELOVL fatty acid elongase 5, *ELOVL6*: ELOVL fatty acid elongase 6, *HSD17B12*: hydroxysteroid 17-beta dehydrogenase 12, *PFKFB1*: 6-phosphofructo-2-kinase/fructose-2,6-biphosphatase 1, *ACACA*: acetyl-CoA carboxylase alpha, *FASN*: fatty acid synthase, *CREB3L3*: cAMP responsive element binding protein 3 like 3, *FOXO1*: forkhead box O1, *LEP*: leptin, *PPP2R1B*: protein phosphatase 2 scaffold subunit Abeta, *CCNA2*: cyclin A2, *LIFR*: LIF receptor subunit alpha, *TNFRSF19*: TNF receptor superfamily member 19, *IFNGR2*: interferon gamma receptor 2, *IL7R*: interleukin 7 receptor, *TNFRSF13*: TNF receptor superfamily member 13, *EPOR*: erythropoietin receptor, *CCR2*: C-C motif chemokine receptor 2, *IL17RB*: interleukin 17 receptor B, *CCR5*: C-C motif chemokine receptor 5, *TGFB1*: transforming growth factor beta 1, *CYP1A1*: cytochrome P450 family 1 subfamily A member 1, *ADCY5*: adenylate cyclase 5, *ALOX5*: arachidonate 5-lipoxygenase, *PSPH*: phosphoserine phosphatase, *SLIT1*: slit guidance ligand 1.

### Validation of mRNAs by qRT-PCR

**Fig 5:**
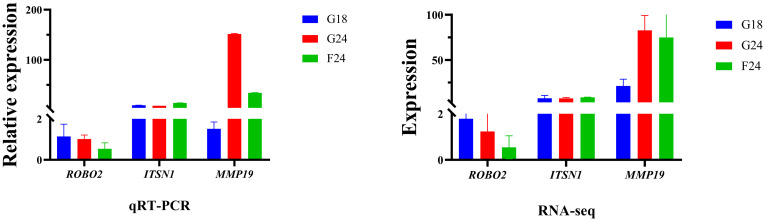
Differently expressed gene validation results.

Validation results by qRT-PCR revealed that the qPCR expression trends of the selected genes were consistent with the results of RNA-Seq analysis, indicating that the transcriptome sequencing method used in this study was accurate and reliable (Fig 5).

## Discussion

As a template to guide protein synthesis, mRNA plays an important role in biological processes [17]. In this study, transcriptome sequencing analysis of mRNA from yak subcutaneous fat tissues under different feeding methods during the withering period revealed differences in the expression of yak subcutaneous fat mRNAs under different feeding methods, with a total of 15,261 known and novel genes identified in the three comparison groups.

The GO function annotation analysis revealed that the DEGs in the three comparison groups were mainly annotated into BP and CC, with a small amount of annotation into MF, which showed that BP and CC were the main biological functions exercised by the DEGs of yak subcutaneous fat in winter with different feeding methods. In the first 20 GO annotation entries of G18_SF vs F24_SF, G18_SF vs G24_SF and F24_SF vs G24_SF, BP mainly includes metabolic process, bioregulation and cellular process, and CC mainly includes cellular part and membrane part, etc. *ACACA* is a complex and multifunctional biotin-containing enzyme, it is an important gene involved in lipid biosynthesis important gene and plays a key role in the regulation of fatty acid synthesis [18]. *ACACA* was significantly down-regulated in G18_SF vs F24_SF and G18_SF vs G24_SF, and up-regulated in F24_SF vs G24_SF, which indicated that the expression of *ACACA* was elevated relative to that of the natural grazing group under the winter housed feeding conditions, suggesting that winter housed feeding yaks favors lipid. Yang C et al. [19] found that the expression of adipogenic genes *ACACA*, *FASN*, *SCD* and *LPL* increased after feeding yaks with high energy diets, and the present study on the increased expression of *ACACA* in yaks after winter feeding was similar to the study by Yang C et al. [19]. Lipoprotein lipase (LPL) is the rate-limiting enzyme involved in lipolysis. The results of Zha et al. [20] showed that there were three genotypes with A as the dominant allele on exon 7 of the *LPL* gene in yak, which plays a decisive role in fat deposition. JEONG et al. [21] studied lipid metabolism related studies in Korean beef cattle and found that up-regulation of the *LPL* gene increased the uptake of fatty acids by adipocyte membranes, which led to an increase in intramuscular fat deposition. This gene was down-regulated in G18_SF vs F24_SF and G18_SF vs G24_SF, and up-regulated in F24_SF vs G24_SF, indicating that the elevated expression of this gene facilitates fat deposition after winter housed-feeding. *ACSL1* (Acyl-CoA synthetase long-chain) is a gene that is involved in the synthesis of lipids and the enzyme involved in the transport and degradation of fatty acids [22]. The *ACSL1* gene is mainly located in the outer mitochondrial membrane, which is a key cellular location for lipid metabolism [23]. It has been reported that ACSL1 is an important factor in whole-body energy metabolism and is associated with triglyceride synthesis and fat deposition [24], and this gene was down-regulated in G18_SF vs F24_SF and G18_SF vs G24_SF, and up-regulated in F24_SF vs G24_SF, which suggests that after winter housetraining, *ACSL1* expression is elevated, which is favorable for triglyceride synthesis and fat deposition. *APOA1* (apolipoprotein A1 protein) is the main component of apolipoprotein A, accounting for 80% of the apolipoprotein A family. *APOA* is mainly responsible for the transport of lipids and proteins [25]. *HDL* (high-density lipoprotein) functions to reverse cholesterol transport and translocate lipids from the peripheral tissues to the liver for catabolism and metabolism [26]. It has been demonstrated that the expression of *APOA1* gene is closely related to the amount of *HDL* [27]. Wang et al. [28] also pointed out that *APOA1* gene is mainly expressed in the liver and kidney of cattle and is associated with intramuscular fat deposition. This gene was significantly up-regulated in G18_SF vs F24_SF, up-regulated in G18_SF vs G24_SF, and down-regulated in F24_SF vs G24_SF, which indicated that the expression of *APOA1*, a related gene that promotes lipid metabolism, was reduced and lipolysis was inhibited after winter housing. Insulin-induced gene (*INSIG*) is an endoplasmic reticulum-synthesized protein that plays an important role in the regulation of cholesterol and lipid anabolism in cells [29]. *INSIG* is mainly annotated to the membrane this term, and the main components of biological membranes are lipids, which are not only involved in the body’s energy supply and storage, but also can be converted into a variety of derivatives involved in metabolic activities [30]. Li et al. [31] found that the expression of *INSIG1* gene was elevated in adipose tissue at the onset of their dietary obesity in Sprague-Dawley rats. This suggests that *INSIG1* gene expression is elevated at elevated adiposity. *INSIG1* gene was significantly down-regulated at G18_SF vs F24_SF and G18_SF vs G24_SF, and up-regulated at F24_SF vs G24_SF, suggesting that the adiposity was elevated after winter housing. Thus, the differences with *ACACA*, *LPL*, and *ACSL1* genes among different comparison groups showed that housed feeding of yaks during the dry period favored fat deposition.

Through the GO annotation of differentially expressed genes and KEGG-enriched pathway analysis, five significantly enriched pathways were screened in this experiment in each comparison group. DEGs are mainly involved in the PPAR signaling pathway, AMPK signaling pathway, fatty acid biosynthesis, ovarian steroidogenesis, and other processes related to lipid metabolism. In this study, we identified the genes *SCD*, *TECR*, *ACACA*, *ELOVL*, *LPL*, and *CYP1A1* as being associated with PPAR signaling pathway, AMPK signaling pathway, lipid biosynthesis, and other processes. The PPAR signaling pathway is a pathway associated with lipid metabolism and adipocyte differentiation [32]. In this study, seven DEGs were significantly enriched in G18_SF vs F24_SF PPAR signaling pathway, in which *SCD* and *LPL* were down-regulated in G18_SF. *SCD* is an endoplasmic reticulum-binding enzyme, which is a key enzyme involved in the biosynthesis of monounsaturated fatty acids [33]. *LPL* is a key rate-limiting enzyme for lipolysis, which was found to favor the deposition of lipids [34]. In this study, *SCD* and *LPL* were significantly down-regulated in G18_SF, indicating that the expression of *SCD* and *LPL* was elevated after feeding yaks during the withering period, and it was hypothesized that *SCD* and *LPL* limited the catabolism of subcutaneous fat in supplementally fed yaks and favored fat deposition. In addition, *LPL* was enriched in Glycerolipid metabolism and Cholesterol metabolism pathway; *SCD* was also enriched in Biosynthesis of unsaturated fatty acids, AMPK signaling pathway and Longevity regulating pathway—worm pathway, in which Glycerolipid metabolism, Cholesterol metabolism, Biosynthesis of unsaturated fatty acids and AMPK, also known as adenylate-activated protein kinase, is a type of adenylate-activated kinase, and AMPK and the AMPK signaling pathway maintain energy homeostasis. AMPK and AMPK signaling pathway can maintain energy homeostasis and play an important role in energy conversion metabolism as a transduction mechanism to ensure the normal physiological mechanism of the cell, and regulate lipid metabolism including fatty acid and cholesterol synthesis by directly phosphorylating proteins or regulating gene transcription in specific tissues (such as liver, adipose, and muscle). In this study, 8 DEGs were significantly enriched in the G18_SF vs G24_SF AMPK signaling pathway, in which *FOXO1*, *LEP*, *FASN*, *ACACA*, and *SCD* were shown to be downregulated, and *PPP2R1B*, *CCNA2*, and *CREB3L3* were shown to be upregulated. The *FOXO1* is the earliest of the FOX subfamily of transcription factors identified to have significant effects on adipocyte differentiation and fat deposition. It has been shown that *FOXO1* is abundantly expressed in adipocytes and is closely related to fat metabolism, among others [35]. In this study, *FOXO1* was down-regulated in G18_SF, and GO annotation revealed that *FOXO1* was mainly involved in positive regulation of apoptosis, positive regulation of gluconeogenesis, and other metabolism-related regulation, from which we hypothesized that elevated expression of *FOXO1* might be related to the role of adipocyte apoptosis and gluconeogenesis in yaks that continued to graze naturally. *PPP2R1B* was less studied in lipid metabolism related studies, but the same family *PPP2R5A* has been proved to be involved in lipid metabolism process [36], *PPP2R1B* was up-regulated in this comparative group, which may be related to lipid metabolism, and its function needs to be further investigated.

## Conclusion

In this study, the subcutaneous adipose tissue of yaks fattened by housed feeding during the dry grass period and free grazing were used to study the expression of their differential genes under different feeding conditions by RNA-Seq technology. Among them, a total of 15,261 expressed genes were identified, 13,959 genes were co-expressed, and 533 DEGs were obtained from differential expression gene analysis. GO annotation and KEGG enrichment analysis of DEGs revealed that, after wither-feeding, the genes related to adipose synthesis in the fat-related PPAR signaling pathway, AMPK signaling pathway, and fatty acid biosynthesis were identified. *SCD*, *ACACA* and *ACSL1* were up-regulated, which indicated that withering feeding was favorable for fat deposition, and these results provided a theoretical basis for subcutaneous fat deposition in yaks.
